# Does citizenship always further Immigrants’ feeling of belonging to the host nation? A study of policies and public attitudes in 14 Western democracies

**DOI:** 10.1186/s40878-017-0050-6

**Published:** 2017-03-01

**Authors:** Kristina Bakkær Simonsen

**Affiliations:** Department of Political Science, Aarhus University, Aarhus, Denmark

**Keywords:** Citizenship, belonging, multilevel regression, conditional effects

## Abstract

Immigrants’ access to citizenship in their country of residence is increasingly debated in Western democracies. It is an underlying premise of these debates that citizenship and national belonging are closely linked, but at the same time there is considerable cross-country variation in how citizenship is approached in Western democracies. In the literature, these differences are typically understood to reflect varying degrees of openness to seeing immigrants as part of the host national community. Motivated by this observation, the article examines whether the degree to which immigrants experience greater attachment to the host nation (i.e. belonging) from having host country citizenship is affected by the host country’s approach to citizenship. This question is analysed with multilevel regressions on survey and country-level data from 14 Western democracies. The findings show that citizenship is associated with increased host national belonging in countries where the host population attaches great importance to citizenship as a mark of national membership, while there is no positive association between citizenship and belonging in countries where the host population considers citizenship less important. Interestingly, citizenship policy does not have a moderating effect on the association between citizenship and national belonging. Implications for future studies of the subjective experience of citizenship are discussed.

## Introduction

Citizenship is the officially sanctioned mark of one’s membership of a political community, of a state. With it come rights and duties and a passport which tells the world where you ‘come from’. While most people have one and the same citizenship throughout life, immigrants may acquire the citizenship of their new country of residence.^1^ As such, the person who was previously an alien is now a fellow citizen, enjoying the same formal status as people who are ‘born’ citizens of the country and who have that citizenship running through their family tree.^2^

When defined as a matter of rights and duties and a passport, citizenship is a purely formal matter of having a certain objective status in a society. However, recent decades’ political debates about the granting of citizenship to immigrants tell another story of citizenship as a highly contested policy domain, which is not just about rules but also about identity. The inflow of immigrants to Western democracies has almost everywhere triggered disputes about who can become a citizen, what should be the demands on people who apply for citizenship, and, ultimately, what it means to be part of our community. These disputes reveal that politicians and public debaters alike often make a close link between citizenship and nationality, between status and identity.^3^

While debates everywhere have it as an underlying premise that being a fellow citizen means being a fellow national, regulation of access to citizenship varies considerably across Western democracies. Countries such as Sweden, Belgium and Portugal maintain that citizenship should be inclusive and fairly easy to obtain, while countries such as Denmark, Switzerland and Spain put greater demands on people who want to become citizens of the country. In the literature on citizenship policy and national identity, such cross-country variations are traditionally seen as reflections of very different conceptions about the boundaries of the national community. Indeed, citizenship policy is taken as either a proxy or an outcome of inclusive versus exclusive national self-understandings (Brubaker, 1992).

Given the contemporary debates about and the very different approaches to citizenship across countries, it seems prudent to examine the possible connections between citizenship and national belonging in the self-understandings of those people who are the target of contemporary debates; immigrants. With reference to the difficulties immigrants face in their attempts to become citizens of their host country, political theorists have taken up questions about the limits of citizenship and the (un)fairness in grounding it in national identity claims. This article will not engage in charging the close linking of citizenship and national identity, but rather take the assumption of such a link as its point of departure. Thus, I examine the idea that immigrants become fellow nationals when they obtain citizenship in the host country. The central concept is national belonging, understood as the subjective feeling of attachment and identification with the nation. The question is whether citizenship and feelings of attachment to the host nation are as closely associated as is the underlying premise in contemporary discourses around the issue. In particular, I am interested in analysing whether the different approaches to citizenship across Western democracies influence how immigrants experience citizenship. In other words, does citizenship have different implications for feelings of national belonging for immigrants to countries like Sweden compared to countries like Denmark?

## Theory

In a basic sense, the questions posed above concern what it means to be a citizen of a country to which you (or, in the case of second-generation immigrants, your parents) did not belong from birth.^4^ As discussed above, both popular and academic discourse assume a close link between citizenship and national identity, to the extent that the two terms are often used interchangeably (Goodman, 2014; Heater, 1999, p. 28).

The growing literature on citizenship policy, which has taken off in recent decades, refers explicitly to discussions about immigrant integration and issues of belonging as a prime motivation for studying cross-country variation (e.g. Ersanilli & Koopmans, 2010; Goodman & Wright, 2015; Hainmueller, Hangartner, & Pietrantuono, 2015; Yang, 1994). Studying the rules governing citizenship acquisition is important, is the premise, because ‘the different possibilities to acquire citizenship will have lasting implications for the long-term integration of immigrants’ (Howard, 2009, p. 8). Most often, however, ‘integration’ is looked at in terms of objective measures of incorporation (e.g. naturalisation rates or socio-economic parity with the native majority population)^5^ rather than immigrants’ subjective experience of citizenship. As Goodman and Wright (2015) argue, there is a lack of studies of ‘the larger questions of effects of civic integration [requirements] on societal integration generally’.^6^ This article contributes to the discussion about the importance of citizenship and citizenship policy by turning to the issue of immigrants’ host national belonging across Western democracies. Belonging is here understood in terms of identification with and feeling of attachment to the nation (Skey, 2013). It is thus both tied to ideas of membership and the feelings of community evoked from this membership (Kannabiran, Vieten, & Yuval-Davis, 2006). Importantly, this notion of belonging underscores that it is something which must be subjectively experienced; it cannot be determined from one’s objective status.

In theorizing about the emotional value which immigrants may derive from host country citizenship, I connect back to the focus of earlier studies of the origins (rather than effects) of citizenship policy (e.g. Brubaker, 1992 and Howard, 2009). The perspective in this literature is that of the host national society, where citizenship is understood as that which qualifies former resident foreigners to be seen, in the eyes of the native majority population, as part of the host nation. Across Western countries, access to citizenship is much more exclusive than residence, the reason being, in Brubaker’s (1992, p. 182) words, that ‘citizenship in a nation-state is inevitably bound up with nationhood and national identity, membership of the state with membership of the nation’. Thus, by granting or withholding citizenship from immigrants, states are not so much acting to protect their territorial borders; rather they are managing the boundaries of national membership (Wimmer, 2013).

In this view, the regulation of citizenship works not only, or primarily, by a territorial and legal economy (investing rights and obligations in the citizenry), but more importantly by a symbolic economy, which valorises citizenship as a mark of one’s belonging to the nation. Non-citizens, even those who have lived and worked in the country for many years, will, in this perspective, still be counted as aliens, foreigners; non-nationals. It is this tying of citizenship policy to identity politics which makes it so ideologically charged (Brubaker, 1992).

The premise thus is that citizenship has the function of turning former ‘others’ into fellow nationals in the eyes of the host population. Working from this assumption, the present study sets out to examine the conditions under which this would spill over to affect immigrants’ feelings of host national belonging. In this effort, I first take one step back to consider the forces through which citizenship could work not only to naturalise but also to ‘nationalise’ immigrants.

First of all, the rights associated with citizenship (‘the legal economy’) are mentioned as a road to greater belonging. While resident aliens are entitled to certain benefits and sometimes to vote at local elections, all countries have reserved important rights, e.g. the right to vote and run for office in national elections, to citizens only (Howard, 2009, p. 5–9). Also the right to take up certain positions in the public sector is restricted to citizens. Thus, citizenship enables participation in significant areas of the host community, in turn making immigrants more familiar with and more committed to the host society. Citizenship also protects from deportation, and thus may provide immigrants with a basic feeling of safety, which in turn could make them more confident in developing feelings of attachment to and identification with the host community.

The second potential road to greater belonging works through citizenship’s symbolic economy (Goodman, 2014, p. 18). Cf. above, citizenship is seen as an important signal to the individual immigrant that (s)he is regarded as a member of the host community, on equal footing with the native population. Often the argument is negatively framed, referring to the ideas inherent in the institution of citizenship that it ‘marks a distinction between members and outsiders’ (Bauböck, 2006, p. 15). Thus, withholding immigrants the right to citizenship may be a powerful tool that can undermine the ability of newcomers to belong (Wright & Bloemraad, 2012, p. 79), and vice versa for immigrants who naturalise.

While the rights-based argument would work more or less in the same way across countries,^7^ there is considerable variation in the way different states approach and frame citizenship policy, with some countries allowing for much easier access into the citizenry than others. One potential implication of this variation is that being a citizen does not carry the same symbolic value–i.e. does not mean the same thing–to naturalised immigrants in countries with very different approaches to the acquisition of citizenship.

The next question then is which approach to citizenship would strengthen (or weaken) its symbolic value in terms of contributing to feelings of host national belonging? In the literature, easier access to citizenship (i.e. fewer and less demanding requirements) is understood to reflect a ‘liberal’, ‘civic’ and ‘inclusive’ national self-understanding as opposed to a ‘restrictive’, ‘ethnic’ and ‘exclusive’ national self-understanding in states with more demanding requirements (Brubaker, 1992; Joppke, 1999; Kohn, 1944). Evidently, a normative judgement is often invested in the use of these labels, with liberal regimes considered preferable from an integration perspective (often referring to the fact that naturalisation rates are higher in these countries, see e.g. Dronkers & Vink, 2012, p. 392). In the context of national belonging, a liberal approach to citizenship is understood to signal to immigrants that *the door* to the national community is open and that immigrants should feel welcome to belong if they want to. This makes for a conditional hypothesis about the symbolic value of citizenship to the individual immigrant:

- H1a: The more liberal the citizenship regime, the greater the positive association between citizenship and feelings of host national belonging.

This hypothesis reflects the normative connotations of the labels attached to more liberal citizenship regimes. In terms of the personal value or meaning of being a citizen, it does, however, seem possible to formulate an expectation which runs counter to H1a. Namely, if the status as citizen is very exclusive, immigrants who secure this status might see it as *a prize*–an utmost symbol of belonging (Bauböck, Ersbøll, Groenendijk, & Waldrauch, 2006, p. 24; Ersanilli & Koopmans, 2010, p. 775). Vice versa, when citizenship is rather easy to acquire in more liberal regimes, being a citizen does not have the same symbolic belongingness value as it does in more demanding regimes. These considerations lead to the second conditional hypothesis:

- H1b: The more demanding the citizenship regime, the greater the positive association between citizenship and feelings of host national belonging.

The two above hypotheses assume that the personal value of citizenship depends on the meaning attached to citizenship in the host country’s citizenship policy. The line of reasoning is that the symbolic content of the policy regime has a signal value which interacts with the naturalised immigrant’s feelings of host national belonging in consequential ways.

*If* it matters to immigrants how citizenship is approached in the host country, there appears to be another source and kind of signal to take into account: Immigrants may not only be attentive to the *meaning* of citizenship embedded in citizenship policy, but also to the *importance* attached to citizenship in the host population. In some countries, host nationals put great emphasis on citizenship as a mark of being part of their nation, while it matters less for the conception of national membership in other populations. For immigrants living in a country where citizenship is seen as a crucial requirement for being one of ‘us’, the belongingness value of being a citizen might be comparatively stronger. Conversely, if the host population does not make a close link between citizenship and national belonging, why should immigrants?

The implications of these considerations is that citizenship status should matter most for immigrants’ feeling of belonging in countries where the host population makes a close link between citizenship and national belonging, i.e. where citizenship marks an important symbolic boundary between ‘us’ and ‘them’. This leads to the study’s final conditional hypothesis, which supplements H1a and H1b:

- H2: The greater the importance attached to citizenship as a mark of national belonging in the host population, the greater the positive association between citizenship and feelings of host national belonging.

To sum up, the three hypotheses expect that the subjective belongingness value of citizenship is conditioned on how citizenship is conceived of in the host country. I have proposed two distinct conditioning sources; policy signals and attitudinal signals. The policy argument concerns the meaning of citizenship; it can be seen as a door to belonging (H1a) or as a prize for completed belonging (H1b). The attitudinal argument (H2) concerns the salience or importance of citizenship; it can be seen as a crucial mark of national membership or as less consequential for drawing the boundary between those who belong and those who do not belong. The next section presents the data, design and measures to test these hypotheses.

## Data and design

In order to examine the theoretical expectations about interactions between personal citizenship status and country-level conditions, I need individual-level data on immigrants, data on citizenship policy, and attitudinal data from members of the host populations. Data on immigrants’ host national belonging, citizenship status and relevant background characteristics stem from the International Social Survey Programme’s (ISSP) National Identity module from 2013. The ISSP consists of random samples of the adult population in the participating countries. Data was collected by national agencies via face-to-face interviews combined with self-completion questionnaires. The analyses are conducted on the 14 Western democracies which participated in the survey (Germany, Great Britain, the United States, Ireland, Norway, Sweden, Spain, France, Portugal, Denmark, Switzerland, Finland, Belgium and Iceland).^8^

While the ISSP was not designed specifically to study immigrants, it does contain immigrant respondents from all the countries included in the survey. Based on information on the citizenship of the respondents’ parents, an immigrant sample is singled out for analysis. A respondent is regarded as immigrant if both parents were non-citizens of the host country at the time of the respondent’s birth.^9^ The effective sample size for the analysis is 1852 respondents, with country samples varying between 25 (Iceland) to 349 (Switzerland) respondents. Note that small country sample sizes do not pose a problem for the estimation of the random effects hierarchical regression model chosen to test the study’s hypotheses (see below for further discussion of the model choice). Namely, even the smallest country sample (Iceland) exceeds the general rule of thumb in the literature to have a sample size larger than 20 observations (e.g. Rabe-Hesketh & Skrondal, 2012).

Considering issues of representativeness, it is possible that the ISSP sample is biased towards the better integrated or more belonging immigrants. For example, immigrants without a minimum of language skills are excluded from participating in the study, since it is conducted in the host national language(s). Also undocumented immigrants and immigrants low in trust of national agencies may generally refrain from participating in a survey. The level of belonging may therefore be overestimated, but this is assumed to apply equally to all countries in the study and would thus not affect the country-comparative conclusions. In addition, the potential oversampling of the better integrated immigrants is likely to decrease the true difference between citizens and non-citizens (as it is plausible that the factors which make immigrants refrain from participating in a survey are more present among non-citizens). As such, the study provides a conservative test of the hypotheses.

The dependent variable, host national belonging, is constructed from the question: ‘How close do you feel to [host country]?’ There are four response categories, ranging from ‘very close’ to ‘not close at all’. By underscoring subjectivity (‘you’) and emotion (‘feel’), the question captures the connotations of identity and attachment which are central to the theoretical belonging concept.

**Fig. 1:**
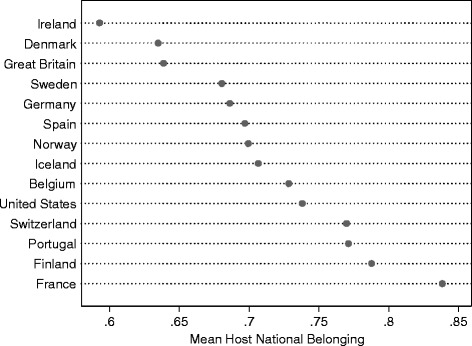
Country mean of immigrants’ host national belonging

Although the variable is ordinal in its original format, I treat is as continuous, since substantial results do not differ between models assuming an ordinal rather than a continuous scale. To ease interpretation of results, I therefore scale the measure to range from 0 to 1 with one indicating the most intense feeling of belonging and zero indicating a lack of host national belonging. The overall mean for the immigrant sample is 0.72. Figure 1 shows the country means on immigrants’ belonging.

The primary individual-level independent variable is a dichotomous measure for whether or not the respondent is a citizen of the country of residence. In relation to this variable, it is relevant to be reminded that the ISSP is not a panel study; it only provides information on belonging and on the citizenship status of the respondent at one point in time (2013). In other words, we cannot examine individual changes in belonging upon acquiring citizenship, but only compare resident aliens and naturalised immigrants. This makes it important to consider how the status as citizen may be confounded with other characteristics which promote belonging. One dimension of the issue is that immigrants must meet certain requirements to obtain citizenship, and these requirements may be positively associated with belonging. The other dimension of the issue has to do with the fact that immigrants are not granted citizenship automatically, but have to apply for it. This corresponds to a problem of self-selection, as it is likely that immigrants who are motivated to apply for citizenship already have a greater sense of belonging to the host nation than immigrants who refrain from applying.

Having made these caveats, recall that the study’s three hypotheses are not primarily concerned with an effect of citizenship on belonging, nor are they formulated in a causal language. Rather, the analysis focuses on the potential of cross-country variations in how citizenship is experienced for naturalised immigrants. The self-selection issue noted above applies to all countries (as only immigrants who want citizenship apply for it), and should therefore not be an issue for the cross-country comparative results. However, the group of naturalised immigrants may differ in composition across countries, following patterns of migration preferences for different migrant groups, and following the pattern of liberal versus restrictive citizenship policy regimes (i.e. some of the immigrants who can become citizens in a liberal regime would not be eligible for citizenship in a more restrictive regime). Therefore the analyses include relevant individual-level variables to control for this issue (see below).

### Citizenship policy

With the growth of the literature on citizenship and integration, indices to measure policies of integration, citizenship and immigration have proliferated. Recently, this has led to academic discussions of content validity, measurement and overlap of different indices, and calls for more careful and theoretically grounded selection of which index to use for empirical analyses of different issues (Goodman, 2015; Helbling, 2013).

**Fig. 2:**
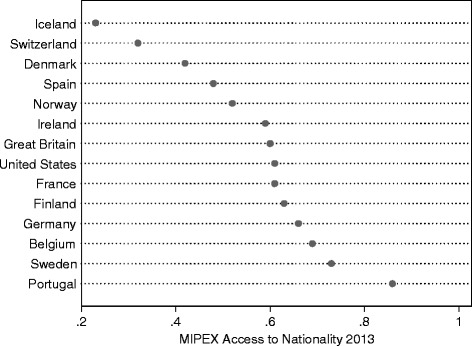
MIPEX Access to Nationality score 2013

I use the Migrant Integration Policy Index’ (MIPEX) Access to Nationality measure for 2013 in the present study because it looks specifically (and narrowly) at citizenship acquisition, and its empirical scope (both in terms of countries and time period) fits the survey data on which this study builds. The index concerns four dimensions of access to citizenship; eligibility (residence requirements and jus soli), conditions (language requirements, integration tests, income/job, application fee level), security of status (discretion versus entitlement to naturalisation), and whether dual citizenship is permitted. Higher scores indicate a policy regime with fewer requirements (i.e. ‘easier’ access). Figure 2 displays country scores on the measure. As can be seen, there is considerable cross-country variation, ranging from a low of .23 for Iceland to a high of .86 for Portugal.

Taking the discussions about index choice into consideration, I perform a robustness check of the results based on the MIPEX measure with an index for ‘Ordinary Naturalization’ from the Citizenship Law Indicators (CITLAW). This index has six dimensions: residence conditions, renunciation requirements (whether dual citizenship is allowed), language conditions, civic and cultural conditions, criminal record conditions, and resource conditions. The correlation of this and the MIPEX index is 0.71, which reveals a great overlap but also differences stemming from the fact that the CITLAW index includes a few more elements. As the MIPEX measure fits slightly better with the timing (2013, against 2011 for CITLAW), and as the CITLAW measure does not cover the US, results for the MIPEX measure are reported in the article, with any differences noted, if the CITLAW measure does not show similar results.

### Citizenship as an attitudinal boundary marker

**Fig. 3:**
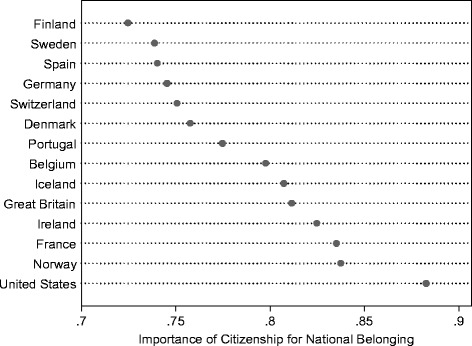
Mean scores for citizenship as a boundary marker in the host population

I utilize the majority sample, i.e. the sample of non-immigrant respondents, to construct the measure of citizenship as a boundary marker. Respondents are asked how important having the country’s citizenship is for being truly Danish (/Swedish/etc.). They are given four response categories from ‘not important at all’ to ‘very important’. I treat the measure as continuous and scale it to vary from 0 to 1, with higher scores indicating greater importance of citizenship as a mark of national belonging. I then construct means of this measure for the non-immigrant respondents in each country to tap the general attitude to the importance of citizenship. Figure 3 shows the country means. As can be seen, citizenship appears to be a rather important marker of national belonging for the majority population in all countries (all country means are well above the midpoint on the measure). There is, however, also considerable variation from a low of .72 in Finland to a high of .88 in the US.

It can be noted that there is no correlation (*r* = 0.00) between the MIPEX measure of citizenship policy and this attitudinal boundary marker measure. Thus, there is empirical support to the theoretical considerations noted above that these two constitute distinct messages about citizenship in the host country.

### Individual-level control variables

There are two main groups of individual-level control variables: (1) the respondent’s country origin and religious identity, and (2) measures of incorporation in the host society. As discussed above, these control variables are included because they may be associated with both citizenship acquisition (through eligibility and motivation, respectively) and with host national belonging. In addition, they may not be evenly distributed across countries. If they are not controlled, we would therefore risk that what appears as country effects are in fact produced by the different compositions of the immigrant population across countries.

Origin country is included as it may affect the ease with which immigrants assimilate into the host culture, and as the conditions in the home country may affect the opportunity structure considered when deciding to apply for host country citizenship (Dronkers & Vink, 2012; Vink, Prokic-Breuer, & Dronkers, 2013). The measure is constructed from information about the country of birth of the respondent’s father,^10^ grouped according to eight world regions (as defined by the UN): EU countries, the rest of Europe, Africa, Asia, the Middle East, North America, Central America, South America, Caribbean, and Oceania.

The next individual-level control is self-identification with a non-Christian religion. As all countries in the study have a Christian heritage, being of another religion is theorised to complicate host national belonging. Religion has also recently been proposed as a factor which may challenge some immigrants’ likelihood of becoming citizens, as questions about gay marriage and church-state relations appear in some citizenship tests (Dronkers & Vink, 2012).

The remaining controls mainly concern the issue that immigrants who are better integrated in the host society in socio-economic terms might also be higher in belonging (Vink, Prokic-Breuer, & Dronkers, 2013; Yang, 1994). This regards years of schooling and labour market incorporation (split into six categories ranging from paid employment to being unemployed or out of the workforce). Finally, sex and age are included as standard controls.

### Country-level control variables

Three country-level control variables are included in the models, as they might affect both the level of belonging in the immigrant population, and the two country-level variables expected to condition the subjective meaning of citizenship (citizenship policy and majority attitudes).

The first control is the size of the immigrant population in the country as a percentage of the total population. Great inflows of immigrants may lead politicians to tighten the access to citizenship or make the population value citizenship less as a criterion of belonging (because it will be seen as increasingly insufficient for ‘true’ belonging). Data stem from the OECD’s databank of the foreign-born population as a proportion of the total population.

The second country-level control variable concerns the country’s employment level. Immigrants’ belonging may decrease in times of economic strain, because it will be harder to gain a foothold in the labour market and society more generally. In addition, several authors refer to economic crises to explain an upsurge in hostility towards immigrants and an increase in nativist feelings (e.g. Crul & Mollenkopf, 2012, p. 4). The greater the economic strain on a country, the greater the expected opposition to immigrants, because immigrants are then seen to be taking ‘our’ jobs (Kesler & Bloemraad, 2010, p. 323). In turn this could lead to a tightening of citizenship policy and/or to de-valorising citizenship as a mark of inclusion. The OECD databank provides data on employment. A score of one indicates full employment.

The final country-level control variable is the level of national belonging in the host population. One could imagine immigrants’ belonging to be either discouraged or encouraged when the surrounding society is very patriotic. At the same time, the host population’s general level of belonging may be associated with the citizenship policy regime and with the importance attached to citizenship as a mark of belonging. The measure is created from taking the country mean value of belonging in the sample of non-immigrant respondents.

### Statistical model

The statistical model used for the analyses is the hierarchical linear regression model with random coefficients. This model choice takes into account the potential issue that observations (immigrants) within the same group (host country) will tend to have correlated outcomes, i.e. that immigrants’ belonging will vary across countries, even when controlled for different distributions of immigrant characteristics (which would violate the OLS assumption of uncorrelated error terms). In addition, the model allows for estimation of country-level effects (i.e. the one component of the interaction terms in the study’s hypotheses). Testing the hierarchical model against an OLS model shows significant variation across countries in immigrants’ belonging (with an intra-class coefficient of .13), which confirms the appropriateness of the model choice.

It should be noted that 14 countries are at the lower end of how many upper-level observations are usually recommended in random effects hierarchical regression models (Rabe-Hesketh & Skrondal, 2012). This means that the estimates may be biased, but this concern is most relevant for the estimation of country-level effects and less so for individual-level effects. In addition, it has been shown that for the estimation of cross-level interaction effects, it is the mean sample size in each group (here: the number of immigrant respondents per country) rather than the number of groups which matters most in terms of statistical power (Mathieu, Aguinis, Culpepper, & Chen, 2012). As the mean immigrant sample size per country (1852 respondents/14 countries = 132) in this study far exceeds recommended minimum levels, this somewhat moderates the issue of having fewer countries in the study than desired. Finally, the risk associated with lower statistical power is to fail to find a statistically significant effect when there is in fact one (type II error). As such, the present study can be considered to provide a conservative test of the hypotheses.

## Analysis

Before the conditional hypotheses are examined, one first question is whether the underlying assumption of a positive association between citizenship and host national belonging finds support in the present data. For this purpose, I run a hierarchical country-fixed effects model, including all the individual-level variables mentioned above. As the fixed effects model controls for all country-level variance, no country-level variables are included. As can be seen from model 1 in Table 1, it does indeed appear that there is a positive association between citizenship and belonging ($\widehat{\beta}$ = 0.06***). Note too that in the random effects model, where country-level variables are included (model 2), the association remains the same, strengthening the belief that the random effects models are correctly specified to test the potential of interaction effects.

**Table 1:** Hierarchical linear regression models, testing the association between citizenship and host national belonging, and potential moderators

	(1) Country fixed effects model	(2) Random effects model	(3) Citizenship policy (MIPEX 2013)	(4) Citizenship as boundary marker
Male	0.01 (0.01)	0.02 (0.01)	0.02 (0.01)	0.02 (0.01)
Age	0.00^***^ (0.00)	0.00^***^ (0.00)	0.00^***^ (0.00)	0.00^***^ (0.00)
Father’s birth country				
EU country	Reference	Reference	Reference	Reference
Other European country	0.08^***^ (0.02)	0.08^***^ (0.02)	0.08^***^ (0.02)	0.08^***^ (0.02)
Africa	0.12^***^ (0.02)	0.14^***^ (0.02)	0.14^***^ (0.02)	0.14^***^ (0.02)
Asia	0.05 (0.03)	0.05 (0.03)	0.05* (0.03)	0.06* (0.03)
Middle East	0.03 (0.04)	0.03 (0.04)	0.03 (0.04)	0.04 (0.04)
North America	−0.02 (0.06)	−0.02 (0.06)	−0.02 (0.06)	−0.03 (0.06)
Central America	0.11 (0.08)	0.12 (0.08)	0.12 (0.08)	0.12 (0.08)
South America	0.06^*^ (0.03)	0.07^*^ (0.03)	0.07^*^ (0.03)	0.07^*^ (0.03)
Caribbean	0.27^***^ (0.08)	0.29^***^ (0.08)	0.29^***^ (0.08)	0.29^***^ (0.08)
Oceania	−0.06 (0.14)	−0.11 (0.14)	−0.11 (0.14)	−0.12 (0.14)
Unknown	0.04 (0.05)	−0.01 (0.02)	0.01 (0.02)	−0.01 (0.02)
Other	−0.22 (0.18)	−0.02 (0.03)	0.02 (0.03)	0.01 (0.03)
Christian	Reference	Reference	Reference	Reference
Non-Christian	0.00 (0.02)	0.01 (0.02)	0.01 (0.02)	0.00 (0.02)
No religion	−0.04^**^ (0.01)	−0.04^*^ (0.01)	−0.04^*^ (0.01)	−0.04^*^ (0.01)
Years of education	−0.00 (0.00)	−0.00 (0.00)	−0.00 (0.00)	−0.00 (0.00)
Paid work	Reference	Reference	Reference	Reference
Unemployed	0.01 (0.02)	0.01 (0.02)	0.01 (0.02)	0.01 (0.02)
Student	0.03 (0.02)	0.03 (0.02)	0.03 (0.02)	0.03 (0.02)
Out of labour market	0.00 (0.02)	0.00 (0.02)	0.00 (0.02)	−0.00 (0.02)
Military service	0.07 (0.17)	0.08 (0.17)	0.09 (0.17)	0.10 (0.17)
Citizen	0.06^***^ (0.01)	0.06^***^ (0.01)	0.05 (0.05)	−0.39 (0.20)
Employment rate		0.12 (0.14)	0.12 (0.14)	0.13 (0.14)
Percentage foreign-born		0.41* (0.18)	0.41^*^ (0.18)	0.43^*^ (0.18)
Host population’s level of belonging		0.44** (0.14)	0.43^**^ (0.14)	0.44^**^ (0.14)
MIPEX Access to Nationality		0.07 (0.07)	0.06 (0.08)	0.09 (0.07)
Citizen*MIPEX Access to Nationality			0.02 (0.08)	
Citizenship as a boundary marker in host population		0.38 (0.22)	0.38 (0.23)	0.13 (0.25)
Citizen*citizenship as a boundary marker				0.57* (0.25)
Constant	0.59^***^ (0.04)	−0.25 (0.23)	−0.25 (0.23)	−0.07 (0.24)

Note: N (respondents): 1852, n (countries): 14. Standard errors in parentheses. ^*^ *p* < 0.05, ^**^ *p* < 0.01, ^***^ *p* < 0.001

The fact that there is a statistically significant and positive association between citizenship and host national belonging tells us that naturalised immigrants ‘on the mean’ feel more attached to the host nation than immigrants without the host country’s citizenship, all else being equal.

From this point of departure, model 3 and model 4 test the expectation that the association between citizenship and belonging is not the same across countries with different citizenship policies, respectively different levels of importance attached to citizenship in the majority population. As there is no correlation between the citizenship policy index and the boundary marker measure, the two variables can safely be included in the same model and thus be controlled for one another.

The first immediate conclusion to be drawn from comparing models 3 and 4 is that the association between citizenship and host national belonging is not conditioned by citizenship policy, while there is a statistically significant and strongly positive effect of the interaction with the attitudinal boundary marker. These results support the idea developed in this article that the subjective meaning of citizenship is not the same across countries–with the qualification that only one of the country conditions suggested appears to moderate the relationship. Note that while the study may provide a conservative test of the hypotheses, the policy interaction effect is far from reaching statistical significance. On the other hand, the fact that the attitudinal interaction effect does reach a level of .05 statistical significance, with a substantially large effect size ($\widehat{\beta}$ = .57*), strengthens the belief that a true moderation exists for this variable.

One may object that since the counter-expectations of H1a and H1b seem equally plausible, the lack of a policy interaction effect may stem from the circumstance that the policy condition is not linear in its effect but instead has an exponential function. I therefore ran the model again with an interaction term of citizenship and the policy measure squared, making it possible to test whether citizenship is e.g. positively associated with belonging at both ends of the citizenship policy continuum, with no (or a smaller) effect at more ‘mixed’ policy positions. The results from this model show no statistically significant effect either ($\widehat{\beta}$(citizenship*MIPEX^2^) = −0.01). Nor does running the same analyses with the CITLAW index (and the CITLAW index squared) yield significant policy effects, which strengthens the belief in this null-finding.

The lack of evidence of a policy interaction effect held up against the positive evidence of an attitudinal interaction effect gives reason to discuss differences between the two country-level characteristics which might explain why one moderates the relationship between citizenship and host national belonging while the other does not. The first difference to be noted is the distinction already made in the theoretical section between the *meaning* and the *importance* attached to citizenship from the host society’s perspective. The results from the present study’s analyses suggest that in evaluating what the status as citizen means for one’s subjective belonging to the host nation, immigrants are affected by the degree to which host nationals think citizenship is important, but not by the exclusivity/inclusivity of the status as citizen. One implication of this finding is that in terms of how immigrants translate citizenship into belonging, it does not seem to matter which signals politicians try to send via their approach to citizenship policy. Or, in other words, citizenship means the same to immigrants in countries with very different approaches to the regulation of citizenship acquisition, all else being equal.

This may be due to the second apparent difference between policies and host national attitudes, namely the difference in the level and form of signals sent by the host country and received by immigrants. While policies are formulated by elites and are formalised in law, host national attitudes are formulated by ordinary people and are informal by nature. One could speculate that immigrants have many more encounters–in the form of everyday interactions and observations of the broader public discourse–with the latter than the former, and should therefore be much more affected by the attitudinal climate in the host society than by formal rules. In addition, there is a difference in the complexity of the signal. In other words, it is one thing to pick up the idea that host nationals think citizenship is important (or not so important), and a completely different thing to be able to decode the, sometimes mixed and complicated, signals embedded in citizenship policies. Indeed, the ambiguity of the meaning of citizenship policy is revealed by the need to formulate two counter-hypotheses in this article. The fact that the policy measure and the attitudinal measure are not correlated (as reported above) strengthens the idea of this interpretation, namely that citizenship policy and the host population’s attitude towards the importance of citizenship are qualitatively different–looking both from the host society and from the immigrant perspective.

**Fig. 4:**
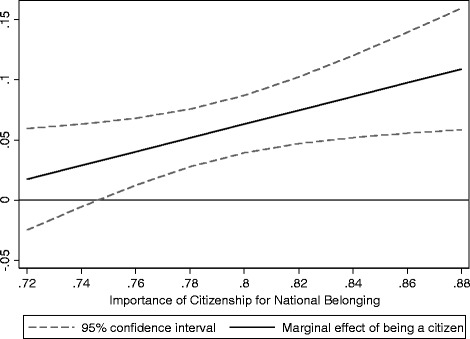
Marginal association of citizenship and host national belonging, over the observed range of the citizenship boundary marker

Having discussed these differences between policies and attitudes, I now turn to a more substantial interpretation of the statistically significant interaction effect of citizenship and the attitudinal boundary marker. The interaction term is, as expected, positive. In other words, the belongingness value, which naturalised immigrants derive from their citizenship, is boosted in nations where the host population attaches great importance to citizenship. This is also illustrated in Fig. 4, which shows the marginal association between citizenship and belonging across the observed range of the attitudinal boundary marker. It can be seen that at the lowest observed levels of importance attached to citizenship (below .75), citizenship’s positive association with host national belonging is no longer statistically significant. As the lowest observed value on the attitudinal boundary marker measure is rather high (well above the midpoint of the scale) this indicates that the host population must think of citizenship as relatively important for it to matter for immigrants’ own ideas about belonging. At the highest observed value on the attitudinal boundary marker measure (the case of the US, cf. Fig. 3), citizenship is associated with a .11 increase in belonging, compared to immigrants without citizenship. This is almost a doubling of the cross-country mean difference between citizens and non-citizens reported in models 1 and 2 in Table 1.

The result thus implies that when (and only when) host nationals think that citizenship is very important for being counted as part of the nation, immigrants also value citizenship as a mark of belonging–and this to increasing degree. On the basis of these data, it appears that the widely assumed link between citizenship and national belonging does not always hold true. Where citizenship is less important for being recognised as one of ‘us’, citizenship does not seem to make a difference for immigrants in terms of belonging. In the present study, this applies to five countries (Finland, Sweden, Spain, Germany and Switzerland, cf. Fig. 3), where the value on the boundary marker measure falls below the .75 threshold. Thus, in these countries, the immigrant with citizenship does not feel greater belonging to the host nation than the immigrant who has not naturalised, all else being equal. The wider implication of this finding is that the positive effects, which citizenship is assumed to have for immigrants’ belonging, depend on the degree to which the surrounding national community sees citizenship as important for national membership.

### Zooming in on the Nordic countries

As this special issue focuses on citizenship and civic integration in the Nordic context, a few final remarks on these countries are warranted. As Fig. 2 in this special issue has shown, there are great differences across the Nordic countries in terms of the requirements attached to the acquisition of citizenship. This also inspired the question posed in the introduction to this article whether citizenship has different implications for feelings of national belonging for immigrants to Sweden compared to Denmark? As Sweden and Denmark are placed at either end on the continuum of citizenship requirements, the seemingly obvious expectation was that it must matter to immigrants’ subjective experience of citizenship. The somewhat surprising answer provided in this article is, however, that it does not seem to do so. Looking only through the lens of citizenship policy, the results imply that citizenship matters equally much (or little) to immigrants’ belonging in Denmark and Sweden, all else being equal. If we turn to the importance attached to citizenship by host nationals, the largest difference within the Nordic context is not between Sweden and Denmark but between Norway and Finland, with boundary marker scores of .84 and .72, respectively (see also Fig. 3). According to the results from the regression model this is a difference which actually matters. While there is no statistically significant association between citizenship and host national belonging at boundary marker scores below .75, the association is highly positive at a level as high as the Norwegian (with a marginal association between citizenship and host national belonging of .10***). As Sweden and Denmark, with boundary marker scores of .74 and .76, respectively, are both close to the tipping point where the association becomes statistically significant, I do not want to make any strong interpretations of the potential differences between these two countries. However, it is safe to say that Norway is the Nordic country whose host population seems to make citizenship matter most to immigrants’ host national belonging–more so than any of the other European countries in this study (only the US has a higher boundary marker score).

## Conclusion

Taking its point of departure in a discussion of the close link made between citizenship and national belonging in popular and academic discourse, this study set out to examine how this link looks in immigrants’ subjective experience. The results support the idea that citizenship matters for feelings of belonging, *but only when it also matters for host nationals* in their perceptions of who belongs. This finding gives reason to believe that the attitudinal milieu is important for immigrants in finding a place in the host national society. The relationship between the host population’s attitudes towards immigrants and immigrants’ own experiences of belonging is an understudied area of research, especially in a cross-national perspective. I think that this finding encourages theorising and further studies on how immigrants pick up the boundary markers valued in the host population and translate them into their personal ideas about belonging.

An equally important result from the study is the null-finding that citizenship policy does not affect how immigrants experience the link between citizenship and belonging. It appears to make no difference to immigrants whether policy makers frame citizenship as a door to integration and belonging, or as a prize immigrants must earn at the end of successful integration. This null-finding resonates with Goodman and Wrights’ study (2015), which finds no statistical evidence to support the idea that tighter citizenship regulations stimulate social integration (in the form of fostering trust and minimising perceptions of discrimination). As they find neither a positive nor a negative effect of civic integration requirements, they conclude that ‘civic integration is more politically strategic and rhetorically popular (particularly with a public hostile to immigration) than it is functional and effective as an integration policy’ (ibid: 18, parentheses in original). In other words, citizenship policy may be effective as a communication tool targeted at the host population, rather than the immigrant population. Indeed, policy makers often frame the regulation of citizenship with reference to conceptions about the boundaries of the national community (Jensen, 2014). As such, they use it to tell a story to the public about who ‘we’ are. This does not mean that the variation in citizenship regimes across Western democracies is unimportant for immigrants–but it seems to matter for other reasons (the access to exclusive rights and political influence, to mention the most obvious) than promoting naturalised immigrants’ belonging.

## Footnotes

1 Depending on the citizenship regime of the host country and of the country of origin, either by renouncing their previous citizenship or by becoming dual citizens.

2 Note, however, that in some countries only ‘natural born citizens’ can become president, thus withholding this right from naturalised citizens.

3 To be sure, debates about citizenship to immigrants also revolve around socio-economic incorporation (e.g. framing language acquisition demands as a requirement to enter the national job market). As this study is specifically interested in the strong assumption about citizenship’s identity dimensions, I will, however, not discuss this aspect further.

4 Note that throughout the article, I use the term ‘immigrant’ to denote both first- and second-generation immigrants.

5 For examples of studies which combine these two objective measures of integration, see DeVoretz & Pivnenko, 2005; Helgertz, Bevelander, & Tegunimataka, 2014 and Pendakur & Bevelander, 2014.

6 As examples of ‘social integration generally’, Goodman and Wright give well-being, political efficacy and sense of belonging–i.e. subjective and ‘deep’ measures of integration.

7 Of course, the status of non-citizens is better in some countries than in others (thus making the ‘gap’ between citizens and non-citizens smaller), but the rights mentioned in the literature as promoters of belonging–the right to vote at national elections and protection from deportation–are everywhere restricted to citizens only.

8 The US stands out as the only non-European country, and it could be argued that it would be reasonable to take it out of the study. Results from analyses performed with and without the US sample included are not substantially different, so the American respondents are kept in the study.

9 Unfortunately, with this operationalisation (and with no information in the data set about the respondents’ birthplace), it is not possible to distinguish between first- and second-generation immigrants. The results from the regression models in this study are therefore mean effects across the two generations.

10 The ISSP also contains information about the birth country of the respondent’s mother, but as almost all respondents have parents from the same world region, I use only father’s birth country in the analyses.
